# Physiological Responses of *Ocimum basilicum*, *Salvia officinalis*, and *Mentha piperita* to Leaf Wounding

**DOI:** 10.3390/plants10051019

**Published:** 2021-05-19

**Authors:** Konstantinos Vrakas, Efterpi Florou, Athanasios Koulopoulos, George Zervoudakis

**Affiliations:** Department of Agriculture, University of Patras, Terma Theodoropoulou, 27200 Amaliada, Greece; kostasvrakas99@gmail.com (K.V.); evteflor@gmail.com (E.F.); tkoulop@upatras.gr (A.K.)

**Keywords:** anthocyanins, chlorophyll, photosynthesis, stress, transpiration

## Abstract

The investigation about the leaf wounding effect on plant physiological procedures and on leaf pigments content will contribute to the understanding of the plants’ responses against this abiotic stress. During the experiment, some physiological parameters such as photosynthesis, transpiration and stomatal conductance as well as the chlorophyll and anthocyanin leaf contents of *Ocimum basilicum*, *Salvia officinalis,* and *Mentha piperita* plants were measured for about 20–40 days. All the measurements were conducted on control and wounded plants while in the latter, they were conducted on both wounded and intact leaves. A wide range of responses was observed in the wounded leaves, that is: (a) immediate decrease of the gas exchange parameters and long-term decrease of almost all the measured variables from *O. basilicum*, (b) immediate but only short-term decrease of the gas exchange parameters and no effect on pigments from *M. piperita*, and (c) no effect on the gas exchange parameters and decrease of the pigments content from *S. officinalis*. Regarding the intact leaves, in general, they exhibited a similar profile with the control ones for all plants. These results imply that the plant response to wounding is a complex phenomenon depending on plant species and the severity of the injury.

## 1. Introduction

Plants are usually exposed to a wide variety of environmental stresses depending on the particular geographic and topographic conditions and the overall plant community of their habitat [1]. These stresses are either abiotic such as water deficiency, high salinity, extreme temperatures, intense radiation [2], or biotic ones such as pathogen infection and insect herbivory [3]. Leaf wounding is a common stress for the plants, caused by both abiotic (e.g., wind) and biotic (insect and animal herbivory) factors [4]. The leaf wounding, even caused by a mechanical reason, not only leads to structural and physiological damage, but can also be a gateway for pathogen invasion.

Thus, plants have developed a wide range of physiological and biochemical mechanisms to cope with leaf wounding [5,6]. First, a lot of locally targeted responses have been observed including enzyme activation, gene and metabolic regulation [5,7], synthesis of signaling molecules [6], and phytochemical reactions concerning several secondary metabolites [8]. These responses lead to quick healing and closure of the wounded tissues even with the activation of mechanisms such as programmed cell death [5].

Moreover, plant leaves often communicate their damage status through metabolites such as jasmonates, which are known regulators of the plant defense, either with leaf-to-leaf signaling [9] or with long-distance signaling, which induces systemic responses against leaf wounding [10]. Leaf wounding also provokes the generation of reactive oxygen species (ROS), such as O_2_^−^ and H_2_O_2_, considering that an accumulation of ROS has been observed a few hours after leaf damage [6,11].

One of the most impressive phenomena is that leaf wounded plants compose airborne signals (e.g., methanol), which not only facilitate the within-plant communication but also the plant-to-plant one, usually inducing the enhancement of the neighboring plants’ defense [3].

All of the above plant responses against leaf damage appear quite fast since they have been observed even in a few hours or days after wounding [6,7,10,11] while jasmonates accumulate within minutes [9].

In this study, we investigated the physiological responses of *Ocimum basilicum* L. (basil), *Salvia officinalis* L. (sage), and *Mentha piperita* L. (peppermint) against leaf wounding. All the aforementioned plants, belonging to the Lamiaceae family, are cultivated in the Mediterranean area and have high economic importance since they have been used from Antiquity as spices, teas, or traditional medicines [12]. In particular, the photosynthetic and transpiration rate, the stomatal conductance as well as the chlorophyll and anthocyanin leaf content were investigated in both control and wounded plants. Moreover, in wounded plants, we measured all the previously mentioned variables on both damaged and intact leaves.

Since all the previous research is limited to the study of short-term (some minutes up to 3–4 days) effects of wounding on plants, our aim was to evaluate the long-term (about 40 days) effect of wounding on the physiological status of different plants and investigate possible differential plant responses.

## 2. Results and Discussion

**Figure 1:**
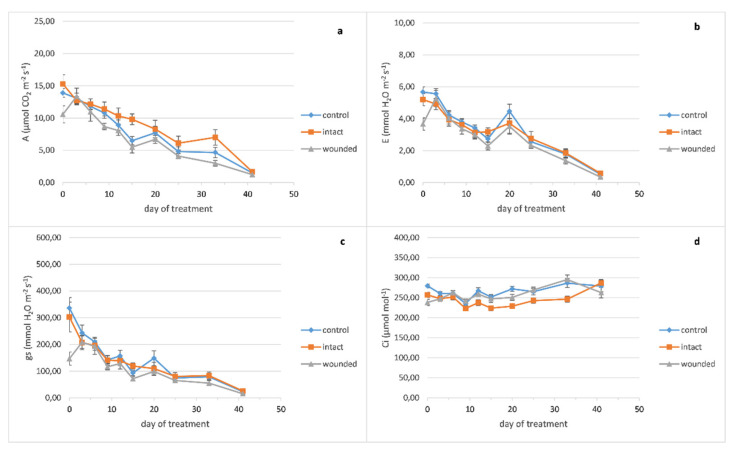
*Ocimum basilicum* leaf gas exchange parameters plotted against days after leaf wounding: (**a**) photosynthetic rate, (**b**) transpiration rate, (**c**) stomatal conductance, (**d**) leaf internal CO_2_ concentration. Data are means (n = 6 or 7) ± standard error of the means (SEM).

The basil wounded leaves exhibited a decrease in the photosynthetic rate compared to both the intact and control leaves (Figure 1a), as expected, since the decrease in carbon assimilation in remaining leaf tissues is usually reported [13]. In particular, the decrease in photosynthesis (31% and 24% compared with the intact and control leaves, respectively) was caused immediately, since it was revealed only one hour after the wounding treatment (zero day), but on the other hand, this decrease was eliminated until the third day. These results are in accordance with previous findings on *Arabidopsis thaliana* (L.) Heynh., which presented a significant decrease of photosynthesis 2 h after the leaf wounding and then its restoration to control levels, 24 h after wounding [7]. Subsequently, the photosynthesis decrease appeared again on the sixth day and was maintained until the end of the experiment. A previous research on imaging photosynthesis (via chlorophyll fluorescence) of wounding leaves demonstrated a reduction in photosynthetic electron transport in regions immediately proximal to the wound compared to more distal ones or corresponding regions of unwounded leaves, one hour after wounding. However, 24 h after wounding, the regions in the vicinity of the wound were capable of photosynthesizing at the same rate as the more distal ones, indicating that they have been fully recovered [14]. These results imply that except from the wounded regions, which are detached or dead, and probably cause a permanent reduction of the leaf photosynthetic capability, the adjacent healthy leaf regions may induce an extra, but transient, loss of photosynthetic rate.

Similarly to the photosynthetic rate results, both transpiration rate (Figure 1b) and stomatal conductance (Figure 1c) of the wounded leaves showed the same immediate decrease of the zero day (of 30–35% for transpiration and 52–57% for stomatal conductance compared with both the intact and control leaves) as has been also described by other researchers for stomatal conductance [15,16], but this decrease had also been eliminated by the third day [16]. The aforementioned decreases of the gas exchange parameters, especially the acute decrease of stomatal conductance, imply that a stomatal limitation related to water loss from the wounded region may cause the photosynthetic reduction although it has been also reported that another possible cause may be the depressed light reaction activity in mesophyll [17]. The strong positive correlation (r ≥ 0.92, Figure 7), according to Pearson coefficient analysis, between photosynthesis, transpiration, and stomatal conductance of basil wounded leaves supports this point of view. These results also indicate that alterations of the gas exchange parameters propagate into remaining undamaged leaf tissue, as was also previously described [18]. After the sixth day, both the transpiration rate and the stomatal conductance of the wounded leaves showed a decrease compared with the intact and control leaves, but this decrease was not as evident as the decrease in the photosynthetic rate.

Furthermore, it was quite impressive that the intact leaves of the wounded plants developed a higher photosynthetic rate not only compared with the wounded ones but also against the leaves of the control plants. Although high photosynthetic rate could be associated with the decreased internal CO_2_ concentration of the leaf, (Figure 1d), this was not confirmed by Pearson analysis (r = −0.25, Figure 7). As it has been reported, the respiration rate of *Arabidopsis thaliana* wounded leaves was decreased 2 h after the treatment, similarly to the observed photosynthesis profile. In contrast, 24 h after wounding while the photosynthesis rate was restored to control levels, the respiration was intensively increased, that is, at least twice as high as the control levels [7]. In general, respiration is increased in wounded plant tissues, implying alterations of the primary metabolism because of the increased energy demand for defense and repair mechanisms [14,19]. Furthermore, it is known that the tissues in the vicinity of the wound increase their sink strength, fulfilling their elevated energy demands by hydrolyzing sucrose and provoking phloem unload [14]. Therefore, the intact leaves of the basil wounded plants may operate as source organs, showing increased photosynthetic rate and thus fulfilling the increased energy and carbon demands of the wounded ones. Concerning the effect of wounding on the basil productivity, upon completion of the experiment, the total dry mass of shoots plus leaves was decreased by 8% for wounded plants compared with the control ones, although their fresh mass and the plant height were unaffected (data not shown).

It is worth noting that the fertilization of the sixteenth day seemed to provoke a transient increase in the gas exchange parameters only at the twentieth day (Figure 1a–c).

**Figure 2:**
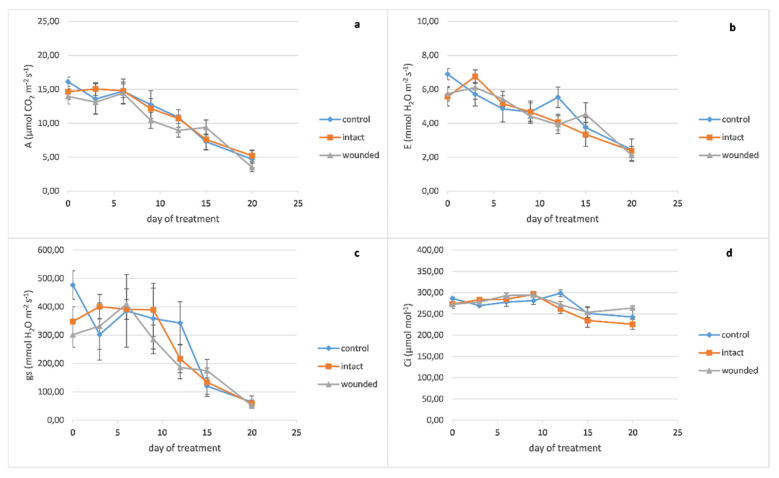
*Mentha piperita* leaf gas exchange parameters plotted against days after leaf wounding: (**a**) photosynthetic rate, (**b**) transpiration rate, (**c**) stomatal conductance, (**d**) leaf internal CO_2_ concentration. Data are means (n = 6 or 7) ± standard error of the means (SEM).

Peppermint wounded leaves revealed a decrease of photosynthesis (Figure 2a), transpiration (Figure 2b), and stomatal conductance (Figure 2c) on day zero, 14%, 17%, and 37%, respectively, compared with the control leaves, like the basil ones. On the other hand, after the sixth day, it seems that there was no substantial decrease in the wounded leaves’ gas exchange parameters, as was revealed in the basil ones. Likewise, the leaf’s internal CO_2_ concentration differences between the different treatments had been diminished (Figure 2d).

**Figure 3:**
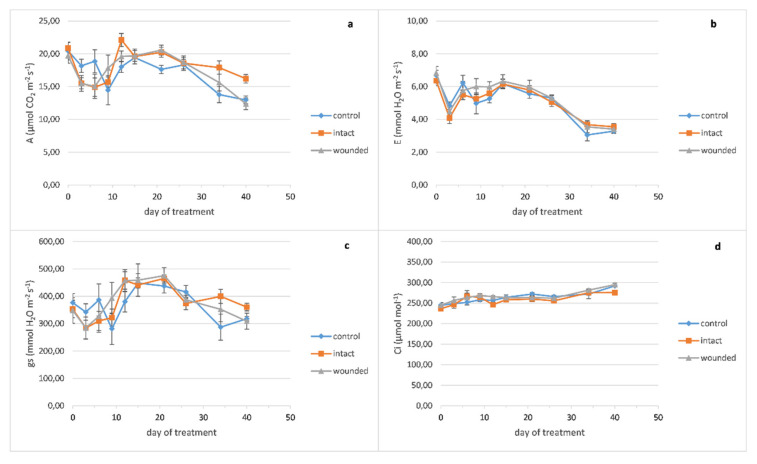
*Salvia officinalis* leaf gas exchange parameters plotted against days after leaf wounding: (**a**) photosynthetic rate, (**b**) transpiration rate, (**c**) stomatal conductance, (**d**) leaf internal CO_2_ concentration. Data are means (n = 6 or 7) ± standard error of the means (SEM).

Concerning the effect of leaf wounding on sage gas exchange parameters, it seems that there are no evident differences between the different treatments throughout the experiment (Figure 3). As a consequence, there was no effect of wounding on the plant productivity upon completion of the experiment, considering that the total fresh and dry mass of shoots plus leaves and the plant height were unaffected (data not shown).

The different responses of the gas exchange parameters between the studied plants may be due to the inherent properties of each species [13] to cope with leaf wounding, as has also been described for other plants [20]. Moreover, the extent of the wounding area may also be a crucial factor since it has been reported that leaf damage of about 2.5% of the total leaf area of strawberry plants do not alter their photosynthesis and transpiration [21]. On the other hand, 10% leaf damage, caused by leafhoppers on sycamore seedlings, induced a significant decrease in both the above parameters immediately after the removal of the leafhoppers, while 16 days later, the photosynthesis had been fully recovered and the transpiration was slightly higher than the control plants. In contrast, the respiration rate of sycamore plants was unaffected [22], although respiration is usually increased after wounding [14,19].

In summary, basil and peppermint indicate that mechanical wounding provoked an immediate decrease in the gas exchange parameters, but a few days later, these parameters were restored to control levels, as has also been previously shown [7,23]. Considering the long-term effect of wounding, our results exhibit a range between decreased (basil) and rather unchanged (peppermint and sage) gas exchange parameters. In general, different plant species exhibit a wide range of decreased, unaffected, or even increased gas exchange parameters after wounding [17,24]. Furthermore, it has been referred that leaf wounding may have a protective effect against another abiotic stress such as chilling, improving the gas exchange parameters of the chilled plants, as was shown for *Triticum aestivum* L. [23]. Therefore, the gas exchange parameters’ response to wounding is a complex phenomenon. Obviously, it depends on both the direct and indirect effects of wounding that is: (a) the reduction of the leaf area and (b) the severed vasculature, the altered sink demand, the defense induced autotoxicity, and the defense induced downregulation of photosynthesis, respectively [18]. Moreover, the plant response is affected by the type of wounding (mechanical injury, insect chewing, phloem sap feeding etc.) [25], while the wounding-induced reduction of photosynthesis is related to decreased concentrations of the light reactions products, ATP and NADPH, implying that a large number of molecules with different connections between them are involved in the plant response [4].

**Figure 4:**
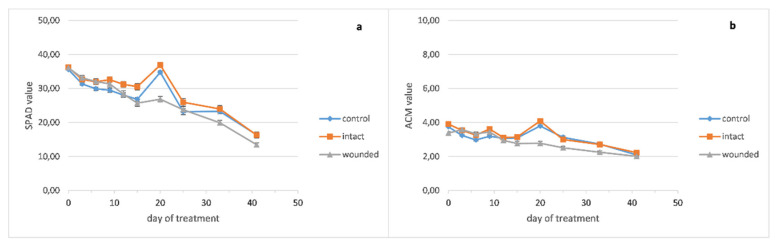
*Ocimum basilicum* leaf pigment content plotted against days after leaf wounding: (**a**) chlorophyll, (**b**) anthocyanins. Data are means (n = 18 or 21) ± standard error of the means (SEM).

**Figure 5:**
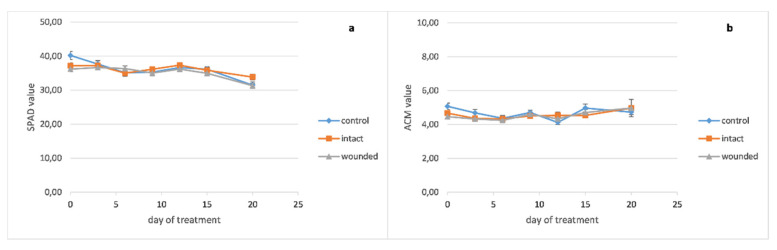
*Mentha piperita* leaf pigments content plotted against days after leaf wounding: (**a**) chlorophyll, (**b**) anthocyanins. Data are means (n = 18 or 21) ± standard error of the means (SEM).

**Figure 6:**
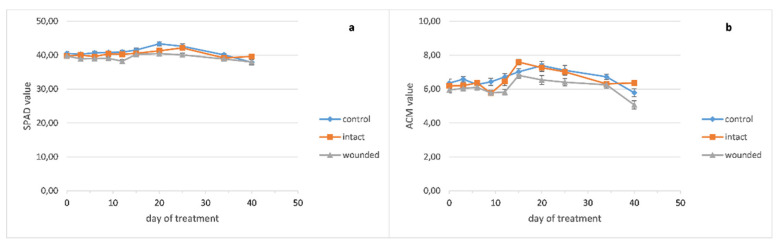
*Salvia officinalis* leaf pigments content plotted against days after leaf wounding: (**a**) chlorophyll, (**b**) anthocyanins. Data are means (n = 18 or 21) ± standard error of the means (SEM).

The chlorophyll content of the basil wounded leaves (Figure 4a) became lower than the intact and control ones around the twelfth day and remained decreased until the end of the treatment. The peppermint leaf chlorophyll content seems to be unaffected from the wounding treatment (Figure 5a), while the chlorophyll content of the sage wounded leaves presented a minor decrease compared with the intact and control ones (Figure 6a).

**Figure 7:**
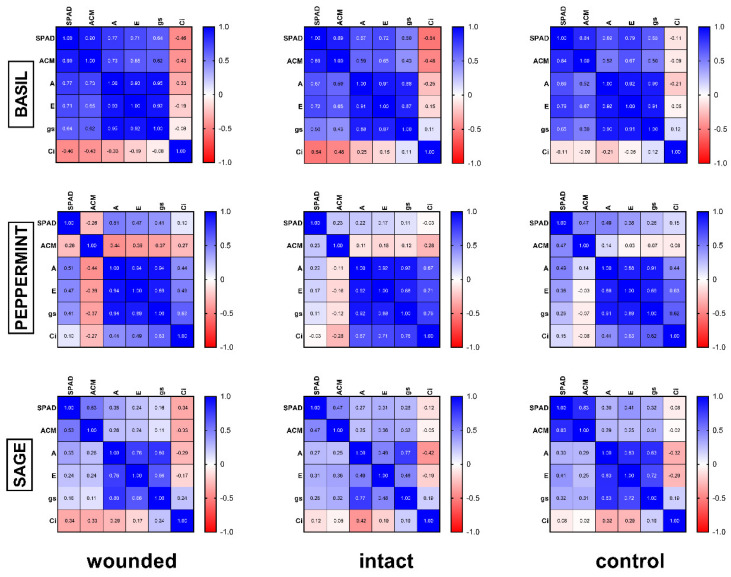
Pearson coefficient analysis heatmap (r values) of the studied plant species and treatments.

It seems that lower chlorophyll content in wounded leaves, as measured in basil (Figure 4a), corresponded to decreased photosynthesis (Figure 1a) and the strong positive correlation (r = 0.77, Figure 7) between them favors this hypothesis. On the other hand, this correlation weakened in peppermint (r = 0.51, Figure 7) and sage (r = 0.35, Figure 7) wounded leaves where chlorophyll content and photosynthetic rate remained rather stable.

Moreover, the pigment contents of the basil wounded leaves seemed to be rather unaffected from the fertilization of the sixteenth day, while the intact and control ones showed a significant but rather transient increase of 20–29% for chlorophyll and 22–30% for anthocyanins, since this increase was eliminated five days later (Figure 4a,b).

Previous studies indicate that, in general, wounding promotes the decrease in leaf chlorophyll content while this effect is also dependent on the wounding intensity [7,26,27]. Furthermore, depending on the type of wounding (hole punching or piercing), the content of the photosynthetic pigments may remain unchanged or even increase [24]. The wounding effect on the leaf chlorophyll content also seems to be related to other environmental conditions, since it has also been reported that under dark conditions, wounding may delay the loss of chlorophyll [28].

The anthocyanin contents of wounded and intact basil leaves became slightly higher than the control ones until the ninth day (Figure 4b). Thenceforth, the content of the wounded leaves became lower until the end of the experiment while the intact and control ones exhibited similar contents. Moreover, against the fertilization of the sixteenth day, the anthocyanins of the basil leaves demonstrated a similar pattern as chlorophyll, that is, unaffected content of wounded leaves and significant but transient increase for the intact and control ones.

In contrast, the anthocyanin contents of peppermint seem to be rather unaffected from the wounding treatment (Figure 5b) while sage wounded leaves developed decreased anthocyanins compared with the intact and control ones (Figure 6b).

It is known that leaf wounding causes anthocyanin biosynthesis and accumulation [8,29,30]. Anthocyanins are not only color providing pigments, but they also contribute to plant resistance against the different biotic or abiotic stresses [29]. Anthocyanins contribute to phytochemical responses against insect herbivory or pathogen infection. Moreover, they can also protect leaf cells from oxidative damage, scavenging a large range of reactive oxygen species, although they may be of secondary antioxidant importance considering that anthocyanins accumulate in the vacuole and not in the cytoplasm or the chloroplasts where the reactive oxygen species are generated. It has been reported that mechanical wounding on young leaves of *Pseudowintera colorata* (Raoul) Dandy induces biosynthesis of anthocyanins, beginning 32 h after wounding and accumulating them since at least 92 h after wounding, from regions approximately three or four cells distant from the necrotic lesion to regions about 1 mm far away. However, the extent of the plant’s response not only depends on the severity of the injury, but also of the leaf age since anthocyanin biosynthesis was not developed in older leaves [8].

Our results exhibited quite different anthocyanin profiles of the wounded leaves, ranging from a typical increase for 3–9 days after wounding and decrease afterward (basil, Figure 4b) to no effect (peppermint, Figure 5b) or even a decrease of anthocyanins from the beginning of the treatment (sage, Figure 6b). In general, these results resemble the corresponding ones of the chlorophyll content. Furthermore, a positive correlation between chlorophyll and anthocyanins, either strong (basil, r = 0.90) or weak (sage, r = 0.53), supports the above results, while on peppermint, where there was no effect on leaf pigments, no correlation was observed (r = −0.26).

Regarding the possible correlation among all variables, the corresponding Pearson analysis (Figure 7) demonstrated: (a) strong positive correlation between photosynthesis, transpiration, and stomatal conductance for all the plant species and treatments, and (b) positive correlation between chlorophyll and anthocyanin content for every treatment on basil and sage, but weak or no correlation on peppermint.

Conclusively, the plant responses to wounding seem to be a complex network of interactions between different physiological functions and molecules. The type and strength of the responses depend on plants species, developmental stage, type and extent of wounding, and environmental conditions. Plants have not only evolved sophisticated mechanisms to cope with wounding effects, but furthermore, some of them may take advantage of mechanical wounding to manage other abiotic stresses such as chilling [23], or even increase their growth rate after wounding [31]. Our results indicate that among the examined plant species, basil is a more sensitive plant against wounding while peppermint and sage are more tolerant.

## 3. Materials and Methods

### 3.1. Plant Material and Experimental Conditions

For each of the studied plant species (*Ocimum basilicum*, *Salvia officinalis*, and *Mentha piperita*), 14 young seedlings of the same height were obtained from a local nursery at the end of July. They were transferred to an experimental field of the University of Patras in Amaliada (South-West Greece, 37°48′ N, 21°21′ E) and transplanted to 4 L pots filled with loamy sand soil. All plants were grown under the same environmental conditions during the whole experiment. The plants were irrigated daily in the afternoon. Each plant was irrigated until there was runoff from the pot. The average daily irrigation dose per plant was 1225 mL for basil, 675 mL for sage, and 880 mL for peppermint. Fertilization was performed twice for all plants before the wounding treatment. Only for basil plants was fertilization also performed on the sixteenth day after the wounding treatment. The fertilization dose each time was 1 gr of crystalline fertilizer 20–20–20 (N–P–K) per plant. The average daily temperature during the experiment ranged from 19.8 °C to 29 °C and the average daily relative humidity ranged from 41.6% to 86.2%.

After 20 days of acclimation and growth period, the seedlings of each species were divided at random into two groups of seven plants per group. One group remained intact and was used as the control of the experiment. The plants of the second group were wounded (day 0) from the top to the bottom leaves of the shoot with a cork borer of 0.6 cm diameter, causing 1–4 holes on each leaf depending on its size without damaging the midrib. The above damaging treatment was conducted only on the leaves of half of the plant shoots, henceforth called wounded leaves, while the leaves of the other half were the intact leaves.

Upon completion of the experiment, about 40 days after wounding, the height of the basil and sage plants was measured. All the plants were harvested and the above-ground part (shoots plus leaves) from each plant was weighed. To obtain the dry weight, the samples were dried to constant weight in an oven at 70 °C for 72 h.

All the below experimental measurements of the leaf gas exchange parameters and the pigment content were conducted for about 40 days for the basil and sage plants. Unfortunately, the peppermint plants were measured only for 20 days because of extended leaf damage caused from herbivore insects, which appeared subsequently. Consequently, the peppermint plants were removed from the experiment because thenceforth they were unreliable.

### 3.2. Leaf Gas Exchange

Nondestructive measurements of photosynthesis, transpiration, stomatal conductance, and leaf internal CO_2_ concentration were obtained with a TARGAS-1 Portable Photosynthesis System (PP Systems, Amesbury, Massachusetts, USA) at 10:30 a.m. on healthy, completely expanded young leaves, that is, on the third or fourth node from the top of the shoot. Considering that during the 40 day experimental period basil plants showed significant growth and development of new leaves, the leaf gas measurements for basil were first conducted on the third or fourth node from the top, but were gradually obtained from lower nodes (even the eighth ones) in order to keep obtaining measurements from wounded leaves and their corresponding intact ones. The same sampling was also followed for the control plants.

All the measurements were conducted on sunny days. Gas exchange measurements were conducted from 6–7 plants per treatment. For each experimental day, two measurements were conducted from each wounded plant, one measurement from a wounded leaf and one from a corresponding intact one, while only one measurement was conducted from each control plant.

The leaf gas exchange measurements of the zero day (day of wounding) were conducted about one hour after the wounding treatment.

### 3.3. Chlorophylls and Anthocyanins

Nondestructive measurements of chlorophylls and anthocyanins were obtained with a SPAD 502DL chlorophyll meter (KONICA MINOLTA, Tokyo, Japan) and ACM-200plus Anthocyanin content meter (ADC BioScientific Ltd., Hoddesdon, UK), respectively.

The same sampling protocol described above for leaf gas exchange measurements was also followed for the determination of chlorophylls and anthocyanins. Leaf pigment measurements were conducted from six to seven plants per treatment. For each experimental day, measurements were conducted from three wounded and three intact leaves of each wounded plant and three leaves of each control one.

### 3.4. Data Analysis

The results for all the measured variables were obtained from six to seven plants per plant species and expressed as the mean ± standard error of mean (SEM).

With regard to the gas exchange parameters, each experimental day one measurement was conducted on each control plant and two measurements (one on the wounded and one on the intact leaf) on each wounded plant.

With regard to the pigment contents, each experimental day three measurements were conducted on each control plant and six measurements (three on wounded and three on intact leaves) on each wounded plant.

All data were plotted using Microsoft Office Excel.

Pearson correlation coefficient analysis was carried out with GraphPad Prism v.9.0 (San Diego, CA, USA).
